# Supplementary material for: Investigating the role of urban vegetation alongside other environmental variables in shaping Aedes albopictus presence and abundance in Montpellier, France
Source: PLoS One. 2025 Nov 12;20(11):e0335793. doi: 10.1371/journal.pone.0335793 (PMC12611129; doi:10.1371/journal.pone.0335793)
Supplement: S4 Table — The mosquito density represents the marginal mean of the number of mosquitoes caught per trap per 24 hours, calculated based on the sampling environment, with the standard error specified (SE). (DOCX) [file pone.0335793.s004.docx]

| **Area** | **Mosquito female/trap/24h (± SE)** | **Mosquito male/trap/24h**  **(± SE)** | ***Ae. albopictus* female/trap/24h**  **(± SE)** | ***Ae. albopictus* male /trap/24h**  **(± SE)** | ***Cx. pipiens* female /trap/24h**  **(± SE)** | ***Cx. pipiens* male /trap/24h**  **(± SE)** | ***Cu. longiareolata* female /trap/24h**  **(± SE)** | ***Cu. longiareolata* male /trap/24h**  **(± SE)** | ***Cu. annulata* male /trap/24h**  **(± SE)** |
| --- | --- | --- | --- | --- | --- | --- | --- | --- | --- |
| PRK-AGL | 8.40 (± 3.18) | 2.58 (± 1.32) | 6.22 (± 3.05) | 1.64 (± 1.39) | 1.07 (± 0.43) | 0.11 (± 0.08) | 0.02 (± 0.02) | 0.02 (± 0.02) | 0 |
| PRK-BOT | 5.61 (± 1.96) | 5.15 (± 2.50) | 2.91 (± 1.36) | 2.10 (± 1.72) | 2.03 (± 0.69) | 1.68 (± 0.58) | 0.02 (± 0.02) | 0.02 (± 0.02) | 0.02 (± 0.02) |
| RES-AGR | 3.90 (± 1.51) | 1.82 (± 0.94) | 3.30 (± 1.64) | 1.18 (± 1.00) | 0.43 (± 0.19) | 0.04 (± 0.04) | 0 | 0 | 0 |
| RES-LEM | 3.83 (± 1.51) | 1.81 (± 0.97) | 3.31 (± 1.68) | 0.92 (± 0.79) | 0.39 (± 0.18) | 0.26 (± 0.15) | 0 | 0.02 (± 0.02) | 0 |
| RES-SOUL | 4.17 (± 1.60) | 1.39 (± 0.73) | 3.29 (± 1.63) | 0.88 (± 0.74) | 0.25 (± 0.13) | 0.04 (± 0.04) | 0.02 (± 0.02) | 0 | 0 |
| IMP-ACA | 5.84 (± 2.67) | 5.35 (± 3.08) | 4.71 (± 2.65) | 3.34 (± 3.04) | 0.48 (± 0.28) | 0.30 (± 0.22) | 0 | 0 | 0 |
| IMP-BIB | 1.08 (± 0.53) | 0.52 (± 0.34) | 0.92 (± 0.57) | 0.26 (±0.2) | 0 | 0.08 (± 0.08) | 0 | 0.03 (± 0.03) | 0 |
| IMP-DID | 0.61 (± 0.34) | 0.04 (± 0.04) | 0.45 (± 0.30) | 0.03 (± 0.03) | 0 | 0 | 0.04 (± 0.04) | 0 | 0 |
| IMP-SCU | 13.7 (± 6.24) | 6.17 (± 3.54) | 5.56 (± 3.14) | 3.94 (± 3.58) | 2.56 (± 1.19) | 0.69(± 0.43) | 0 | 0 | 0 |

The mosquito density represents the marginal mean of the number of mosquitoes caught per trap per 24 hours, calculated based on the sampling environment, with the standard error specified (SE).
