# Supplementary figures and images for: Investigating the role of urban vegetation alongside other environmental variables in shaping Aedes albopictus presence and abundance in Montpellier, France
Source: PLoS One. 2025 Nov 12;20(11):e0335793. doi: 10.1371/journal.pone.0335793 (PMC12611129; doi:10.1371/journal.pone.0335793)

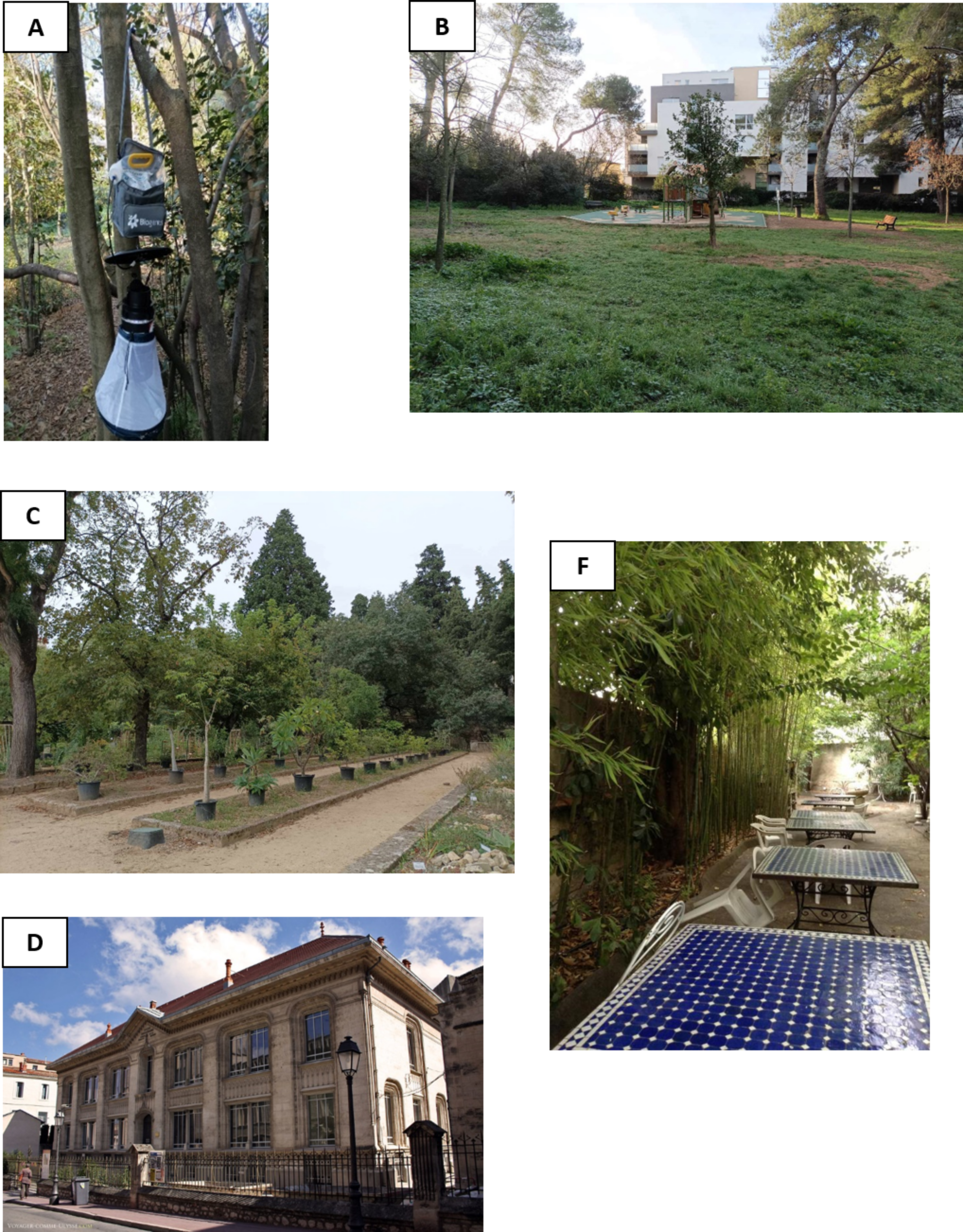

Supplement: S1 Fig — S1A Fig represents a BG-Pro on the field, S1B Fig the Park of Aiguelongue, S1C the Botanical Garden, S1D The Bouisson Bertrand Institute and 1E the Acapulco Hostel. (TIFF) [file pone.0335793.s006.tiff]

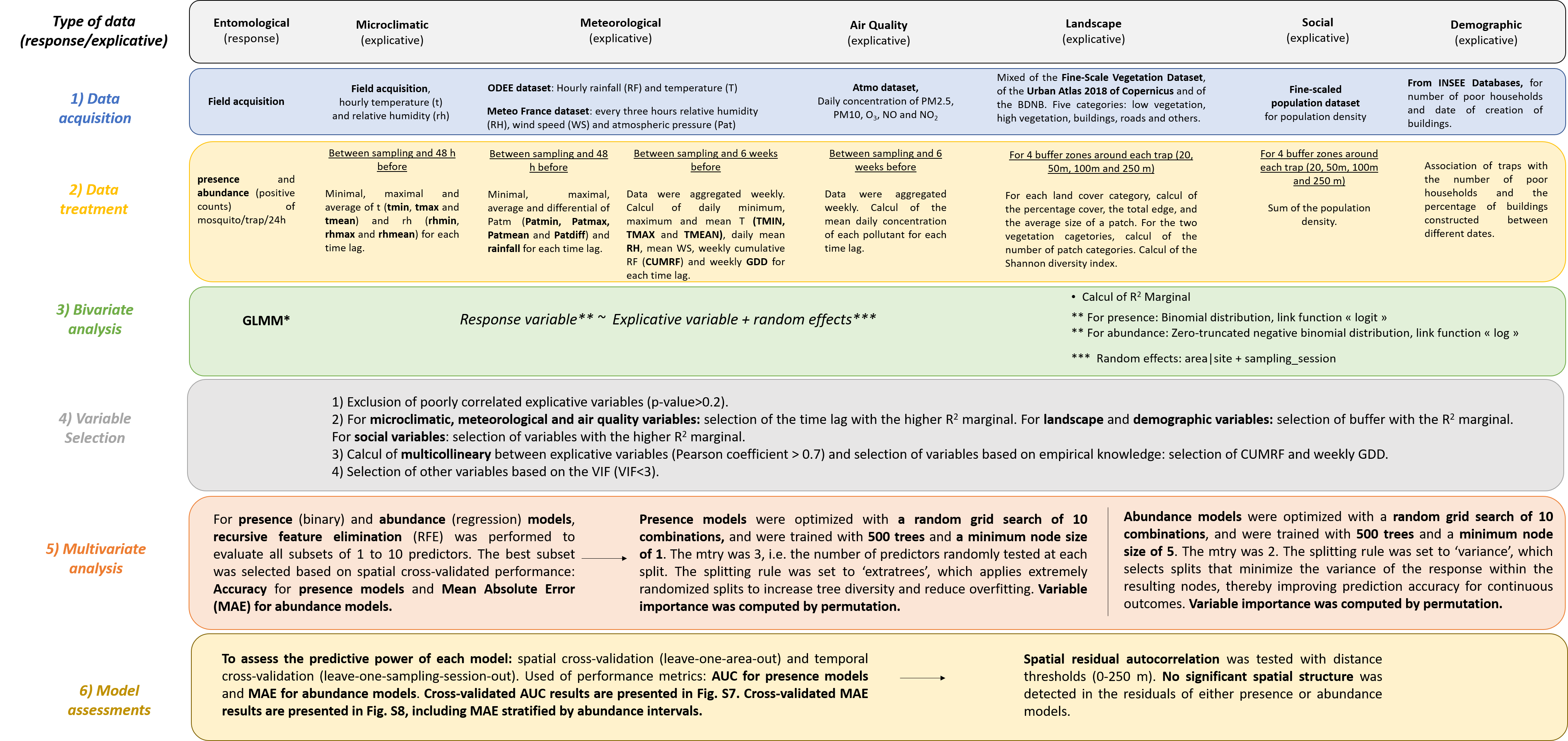

Supplement: S2 Fig — (TIF) [file pone.0335793.s007.tif]

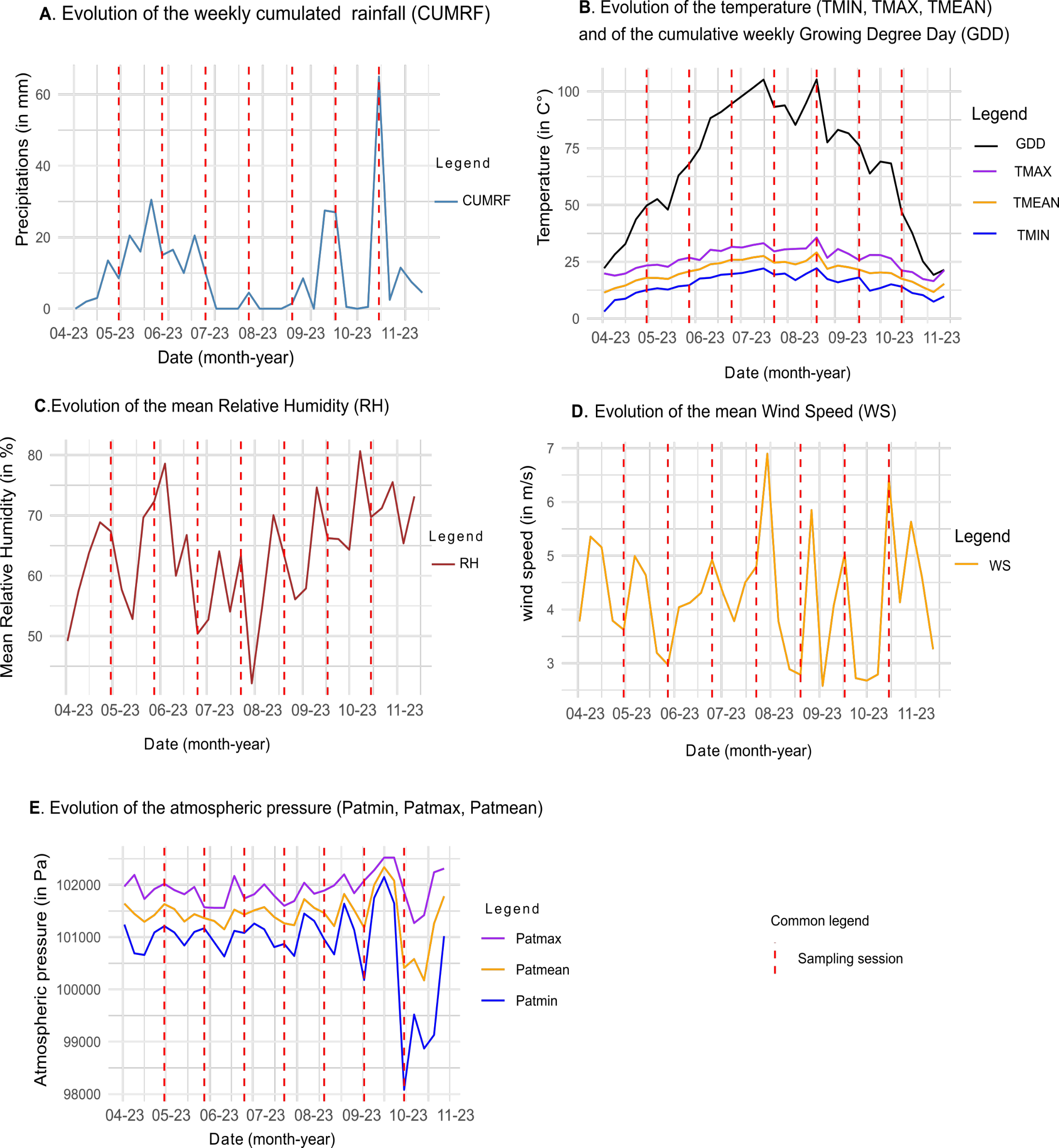

Supplement: S3 Fig — Vertical dotted red lines indicate monthly mosquito sampling sessions. (TIFF) [file pone.0335793.s008.tiff]

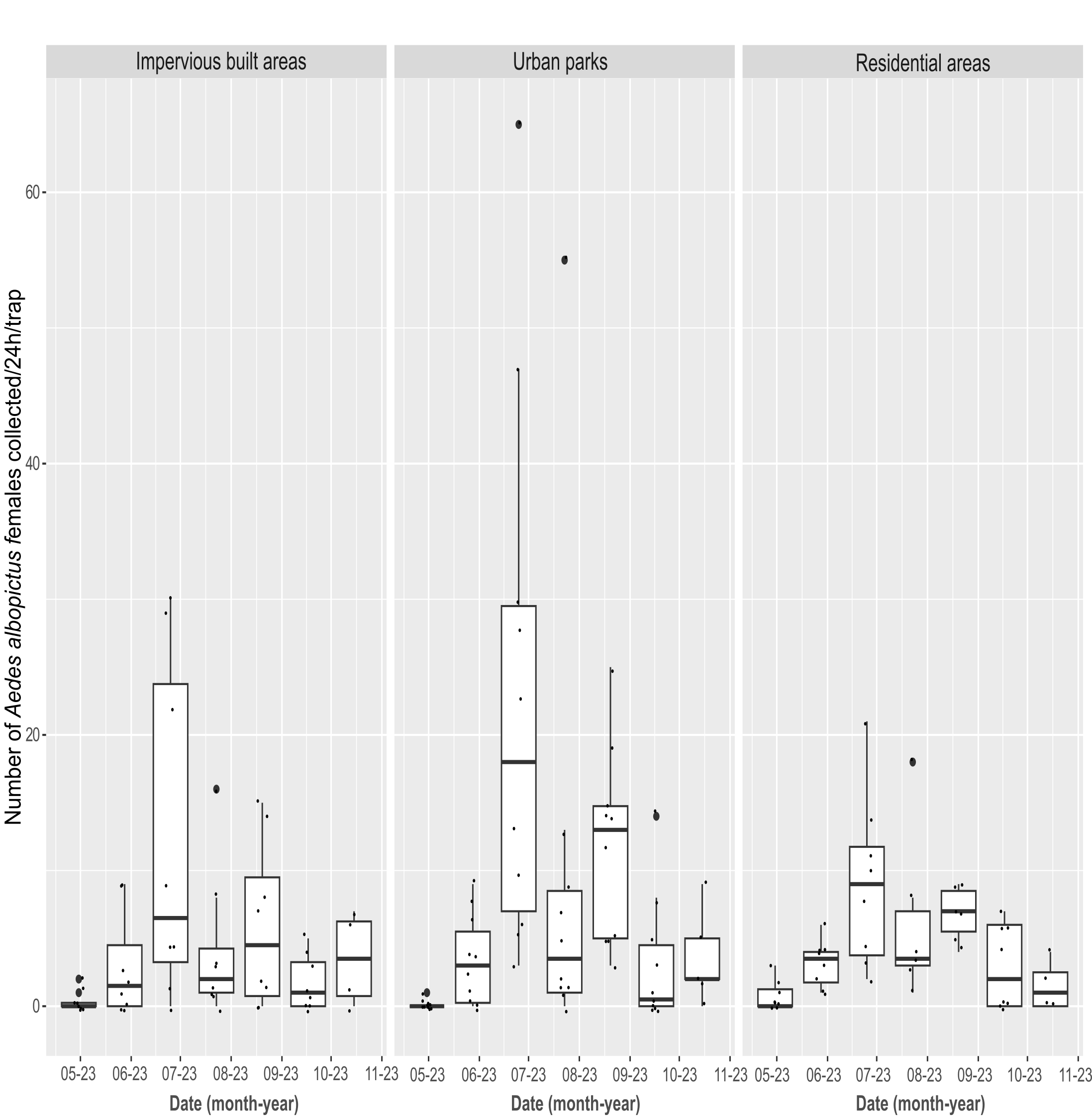

Supplement: S4 Fig — Boxes represent medians and interquartile ranges, whiskers indicate minimum and maximum values, and points show the abundance for each sampling session and day, for each trap, within the three environments. (TIFF) [file pone.0335793.s009.tiff]

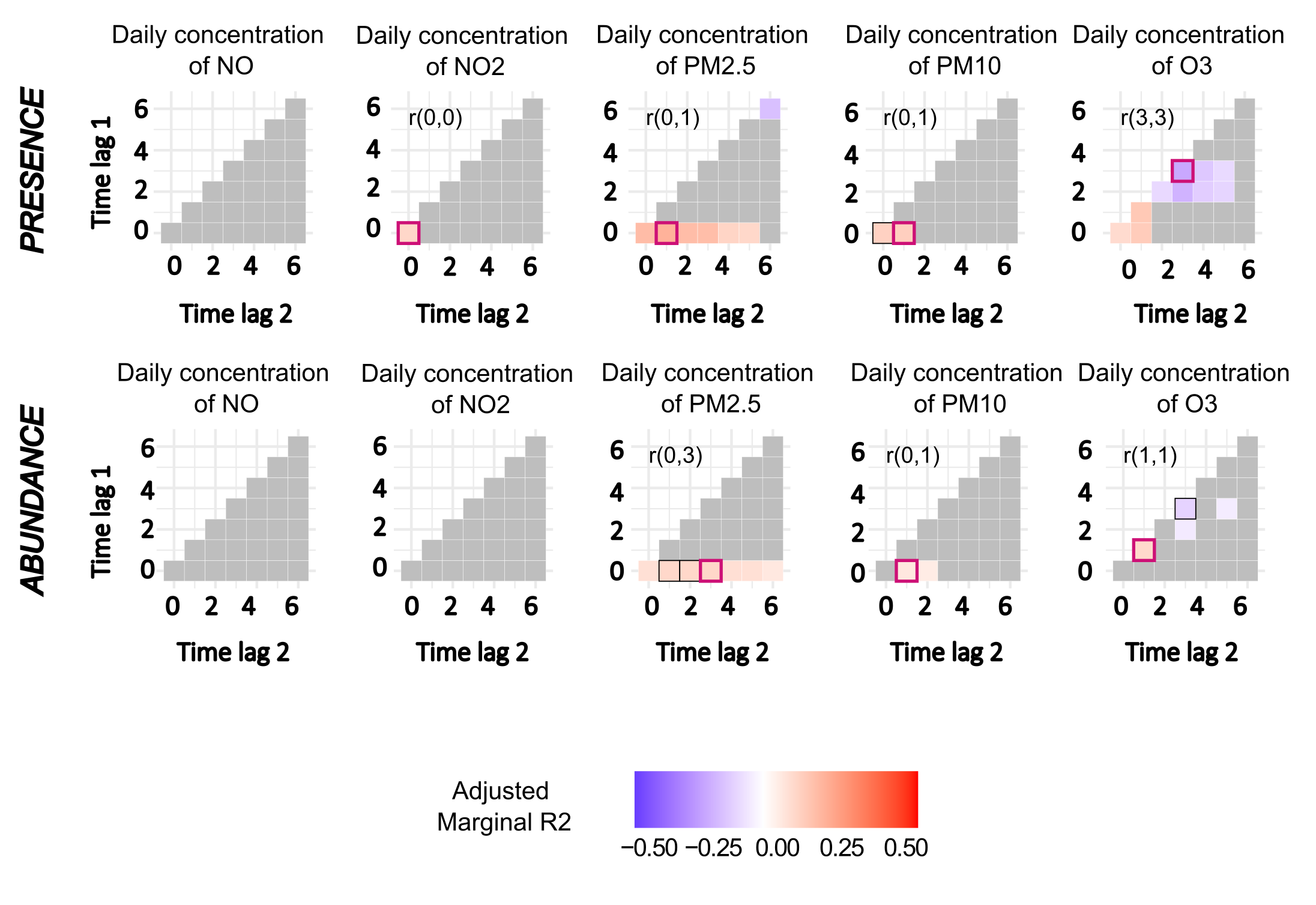

Supplement: S5 Fig — Lagged air-quality variables include daily concentration of nitrogen monoxide (NO), of nitrogen dioxide (NO2), of particulate matter (PM10, PM2.5) and of ozone (O3). Time lags are expressed in weeks prior to sampling. The adjusted marginal R2 reflects the variance explained by the explanatory variable, adjusted for correlation direction. Red-bordered squares highlight the time lag with the highest marginal R2, with the interval indicated in the top left corner. Black-bordered squares denote marginal R2 values close to the highest (within 10%). Gray squares represent correlations with p-values > 0.2. (TIFF) [file pone.0335793.s010.tiff]

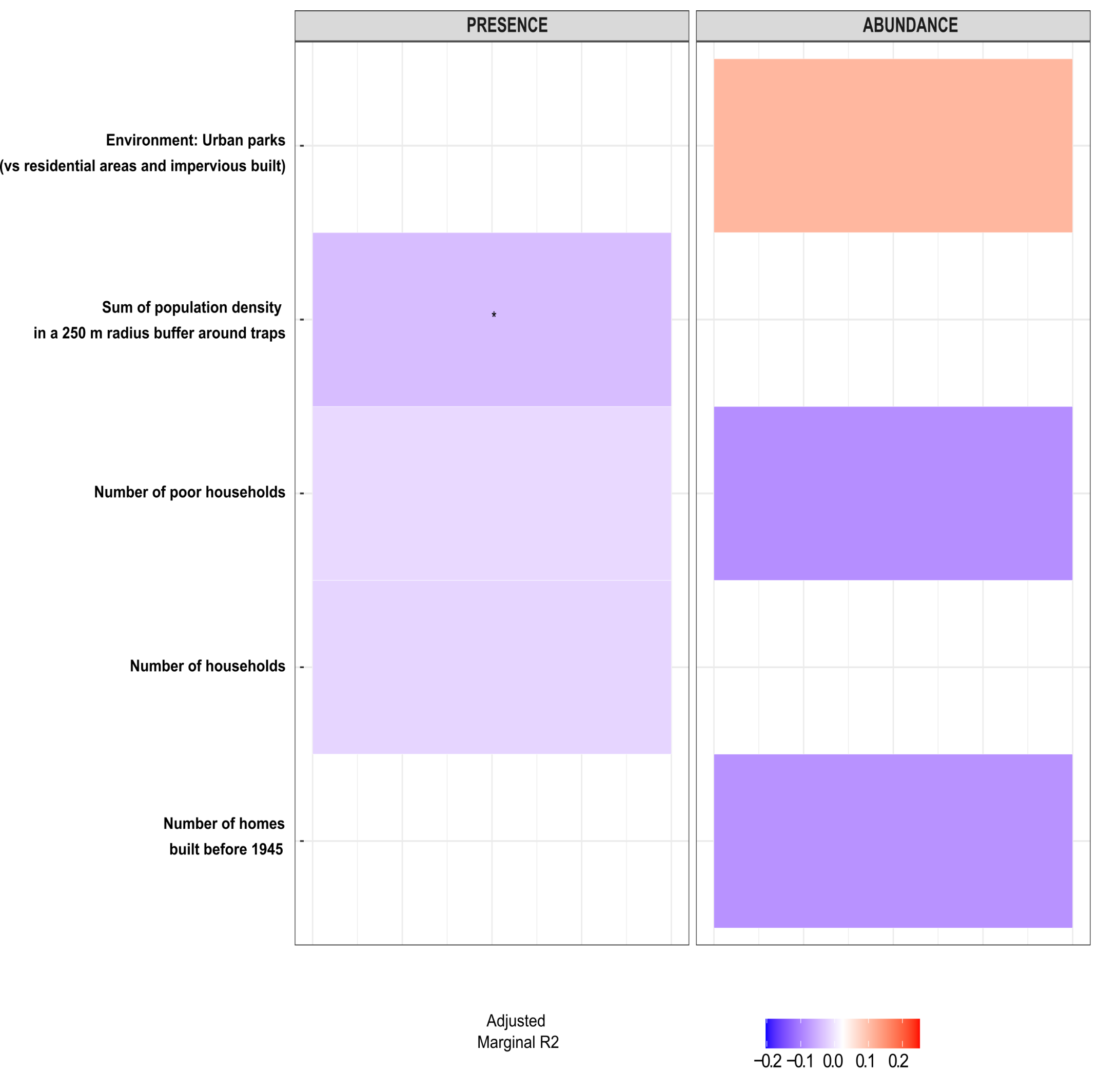

Supplement: S6 Fig — The adjusted marginal R2 reflects the variance explained by the explanatory variable, adjusted for correlation direction. Boxes are colored if the p-value was < 0.2 (No asterisk: p-value ∈ [0.05; 0.2], *: p-value ∈ [0.01, 0.05], **: p-value ∈ [0.001; 0.01]; ***: p-value ∈ [0; 0.001]). Box color depends on the direction of the relationship (blue: negative, red: positive). Color intensity varies according to the marginal R2 value. (TIFF) [file pone.0335793.s011.tiff]

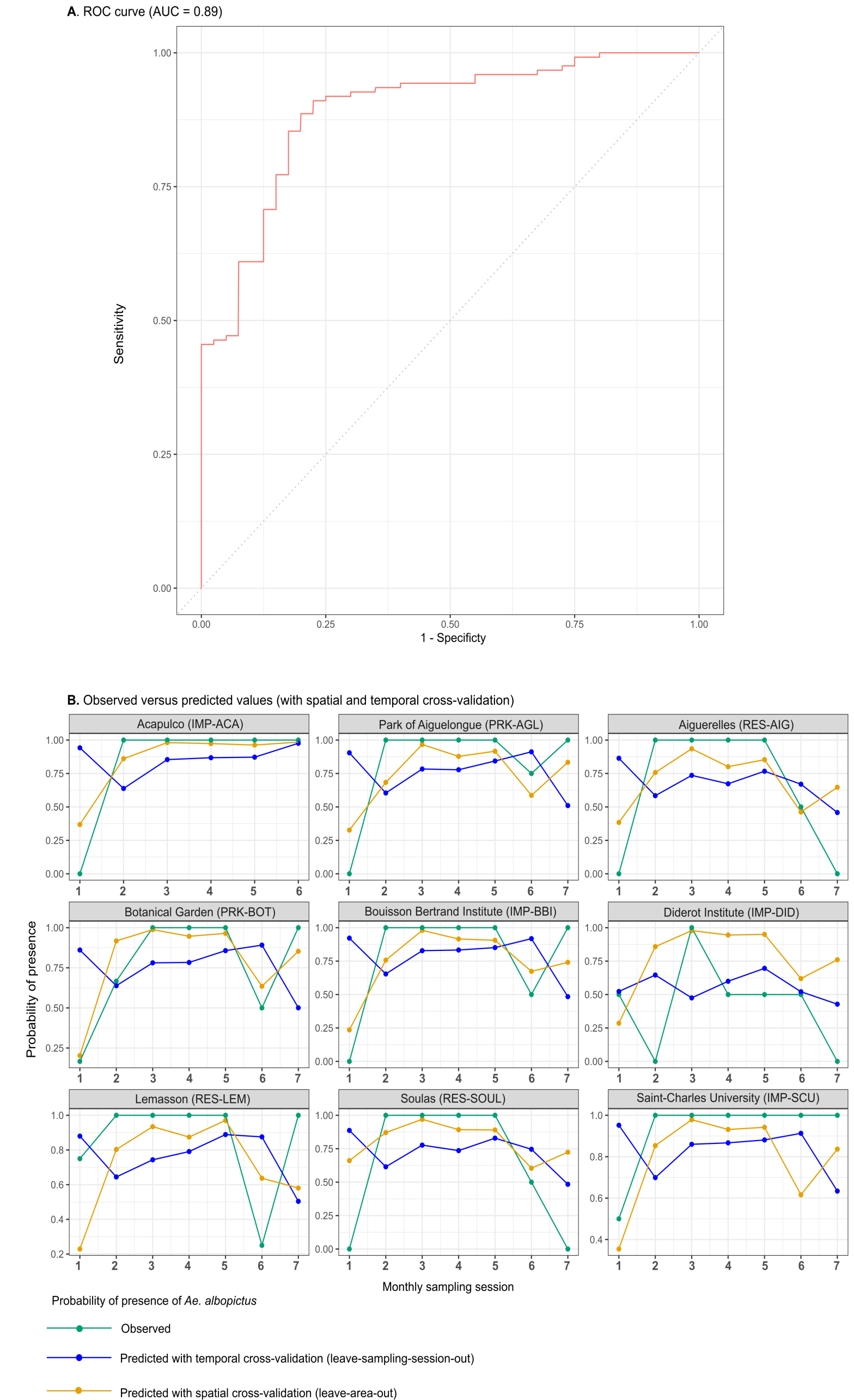

Supplement: S7 Fig — S7A Fig is the Receiver Operating Characteristics (ROC) Curve for the presence model. It illustrates the model’s sensitivity (its ability to predict the presence of Ae. albopictus) against 1 – Specificity (its ability to detect the absence of Ae. albopictus). The Area Under the Curve (AUC) represents the model’s ability to discriminate between the presence and absence of the mosquito, with values ranging from 0 to 1. The closer the AUC is to 1, the better the model’s predictive performance. S7A Fig shows that the model demonstrated good predictive accuracy, with an AUC of 0.89. S7B Fig compares the observed (in green) versus predicted presence probabilities for each out-of-sample leave-area (yellow) and for each out-of-sample leave-sampling-session (in blue). The y-axis represents the probability of Ae. albopictus presence, while the x-axis corresponds to the sampling session. Fig 7B indicates that the model accurately predicted the spatiotemporal trends of presence/absence, although it tended to overestimate presence. (TIFF) [file pone.0335793.s012.tiff]

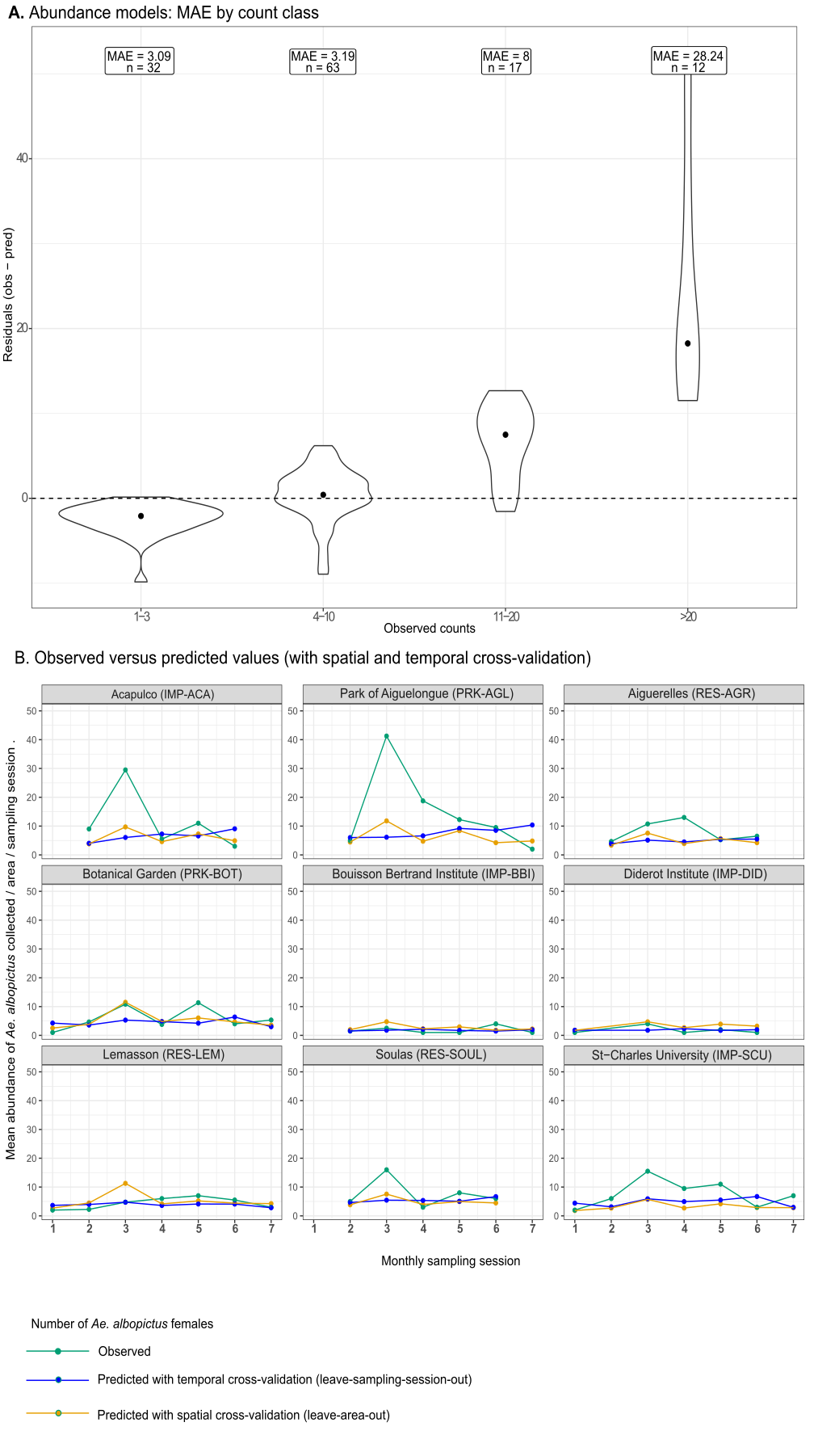

Supplement: S8 Fig — S8A Fig is a violin plot showing the distribution of residuals for the abundance model. The black dots indicate the median values. The Mean Absolute Error (MAE) and the number of observations (n) are displayed in a square above the plot. This figure demonstrates that the model tends to overestimate low counts of Aedes albopictus (fewer than three caught in 24 hours per trap), accurately estimates counts between 4 and 20, and underestimates higher counts (more than 20). S8B Fig compares the observed versus predicted numbers of Ae. albopictus caught per site per sampling session (in green), versus predicted numbers for each out-of-sample leave-area (yellow) and for each out-of-sample leave-sampling-session (in blue). The y-axis represents the mean number of d Ae. albopictus females caught per trap per area per sampling session, while the x-axis represents the sampling session. This figure confirms the observations made in S8A Fig. (TIFF) [file pone.0335793.s013.tiff]
