# Supplementary material for: Investigating the role of urban vegetation alongside other environmental variables in shaping Aedes albopictus presence and abundance in Montpellier, France
Source: PLoS One. 2025 Nov 12;20(11):e0335793. doi: 10.1371/journal.pone.0335793 (PMC12611129; doi:10.1371/journal.pone.0335793)
Supplement: Attachment — Submitted filename: plos_review.docx [file pone.0335793.s015.docx]

**ABSTRACT**

**Line 31**: I was wondering why the term *“impervious”* is used in the context of urban environments. Is it related to the *imperviousness* variable, in the sense that a more impervious environment indicates a higher degree of urbanisation?

**Line 32**: It may be helpful to specify more clearly how on-site microclimate was measured.

**INTRODUCTION**

The transition between urbanisation and its description, and the subsequent discussion on re-greening, is not entirely smooth.

Overall, the introduction conveys the main concepts effectively, and the study objective is clear.

**Line 75**: I do not fully understand how *social disparities* are linked to urbanisation. Does urbanisation increase these disparities, which in turn exacerbate the risk of mental health issues? This connection could be explained more explicitly.

**MATERIALS & METHODS**

Overall, the section requires revision to enhance clarity for the reader. It is not entirely clear which temporal lags were used, nor whether there is spatial aggregation of the data, and at what resolution (e.g. land cover). Spatial resolution is reported only for infrastructure age and low-income household data. Was this resolution (200 m grid) maintained, or were datasets at different resolutions (to be specified) intersected with trap locations and buffers, with values then assigned accordingly? This should be clarified.

Also, It is not clear whether potential vector control interventions (e.g. insecticide spraying or larvicide treatments conducted by the municipality in urban green areas) were monitored or considered during the study period. Such interventions could strongly influence mosquito abundance and presence, and therefore it would be important to clarify whether information on disinfestation activities was collected and how this factor was handled in the analysis.

Additionally, while the general modeling workflow is described, the supplementary materials could be expanded to include more technical details on the statistical analyses. This could include a detailed diagram of the variable selection and modeling workflow, specifications of the GLMM and random forest models (e.g., distributions, link functions, hyperparameters), cross-validation results (spatial and temporal), diagnostics of model performance and residual spatial autocorrelation, and sensitivity analyses regarding buffer sizes and temporal lags. Providing these details would improve transparency, reproducibility, and the reader’s ability to assess the robustness of the results.

**Line 137**: Replace “were vegetated” with “were vegetated areas” or “were covered with vegetation”.

**Lines 168–172**: It is not clear whether one 48-hour trapping session per month was conducted, or whether multiple 48-hour sessions took place within each month. From the results it appears there was one per month per site, which should equal 7 × 15 collections. It is also unclear whether mosquitoes were collected each morning during the 48 hours (i.e. twice per sampling event) or only once after the full 48 hours.

**Lines 192–194**: A description of land cover classes, either in the draft or in the Supplementary Materials, would be useful. The definitions of *percentage of cover*, *total edge length*, and *average patch size* should also be provided, along with details on how they were calculated.

**Line 195**: Provide a definition of the Shannon index.

**Line 204**: The shift from 24 h to 48 h lags is unclear. Perhaps it would be clearer to write: *“for 24-hour periods starting from 48 hours prior to the beginning of each sampling session until its end”*.

**Line 208**: Similarly, I would suggest: *“24-hour periods prior to and during the sampling sessions …”*. Does “as for datalogger data” mean that microclimate was measured with the datalogger and values were taken exactly as recorded?

**Lines 206–209**: Which rainfall dataset was used—the hourly data from the Observatoire Départemental de l’Eau et de l’Environnement (ODEE) station, or from the datalogger?

**Line 215**: *“All weather data were aggregated for the sampling week and each of the six weeks before sampling”*. Does this mean the temporal resolution is weekly, or were different lags created (e.g. weekly mean temperature, two-week mean, daily, two-day, etc.)? Please clarify how the temporal aggregation was performed for the models.

**Lines 218–220**: This reads more like material for the Introduction or Discussion. I would recommend moving the justification for the variable choice after its description and derivation.

**Lines 228–231**: Same comment as for lines 218–220.

**Lines 241–244**: Please specify the years for these data.

**Line 246**: Are the population data from 2016? Are more recent datasets available?

**RESULTS**

In general, the results are presented clearly and logically.

**Line 334 – Table 1**: Should be written *Cs. longiareolata* and *Cs. annulata*.

**Line 427**: Typo: “associtaion” → “association”.

**Line 476**: Since population density is discussed here, the Materials and Methods should specify how it was calculated.

**DISCUSSION**

Overall, the Discussion is solid and compares the findings with previous studies. However, there is limited effort to define how the results could inform practical urban planning (e.g. optimal dimensions of green areas, vegetation management in residential zones, trade-offs between ecological benefits and vector-related risks). Strengthening this aspect would considerably enhance the impact of the paper.

**FIGURES**

**Figure 1**: Panel B lacks a scale bar, and a north arrow would be useful. In addition, the outline colours of sampling environments are difficult to distinguish from the land cover map, which uses similar colours.

**Figure 6**: The legend does not indicate the meaning of the asterisk.

**FUTURE PROSPECTS AND GENERAL THOUGHTS**

This is a very interesting study, particularly in the framework of Agenda 2030 and in reconsidering urban land use. In the context of rising temperatures and increasing urbanisation, the study may pave the way for targeted investigations aimed at integrating documented scientific evidence into urban planning. At present, such planning is often undertaken without considering the loss of ecosystem services or the implications of invasive species that may thrive in anthropogenic environments, particularly those of public health relevance.

However, there are issues concerning the Materials and Methods section, and, as also noted in the text, the draft relies on a single year of sampling. I encourage the authors to consider publication only after collecting at least one additional year of data, which would strengthen the analyses, allow more robust conclusions, and provide a solid foundation for concrete contributions to urban green planning.

I also recommend consulting the following preprint, which addresses a very similar and directly relevant topic and may provide valuable insights for this study and future work:
*Mercat M, Bartholomée C, Fournet F, Alcover Amengual M, Bourquia M, Bouhsira E, Cornel A, Fernandez Cassi X, Fontenille D, Ibáñez-Justicia A, Marti R, Moiroux N, Niang EH, Smith W, Spitzen J, Visser TM, Koenraadt CJM, Simard F. Green cities and the risk for vector-borne disease transmission for humans and animals: a scoping review. bioRxiv 2025.04.01.646559; doi:* [*https://doi.org/10.1101/2025.04.01.646559*](https://doi.org/10.1101/2025.04.01.646559?utm_source=chatgpt.com)

It would also have been valuable to record not only microclimate but also vegetation type at each sampling site (i.e. plant species present). In future studies, it could be investigated which plant species have the greatest influence on mosquito presence and abundance, as suggested by previous work (e.g. Samson et al. 2014). Such knowledge could provide urban planners and green-space managers with practical guidance on plant species selection to reduce mosquito populations.

Finally, in future studies, it would be interesting to evaluate whether arbovirus cases occurred in areas characterised by variables positively associated with mosquito abundance and presence identified in this study, or conversely in areas with features negatively associated with these outcomes.
