# Supplementary material for: Investigating the role of urban vegetation alongside other environmental variables in shaping Aedes albopictus presence and abundance in Montpellier, France
Source: PLoS One. 2025 Nov 12;20(11):e0335793. doi: 10.1371/journal.pone.0335793 (PMC12611129; doi:10.1371/journal.pone.0335793)
Supplement: Attachment — Submitted filename: Response to reviewer.docx [file pone.0335793.s017.docx]

**Dear Editor and Reviewers,**

We are pleased to submit a revised version of our manuscript entitled “Investigating the role of urban vegetation alongside other environmental variables in shaping Aedes albopictus presence and abundance in Montpellier, France” (ID: PONE-D-25-17422). We sincerely thank you for your thoughtful and constructive comments, which have greatly helped us to improve the clarity, accuracy, and overall quality of the manuscript. A detailed, point-by-point response is provided in the following document.

In response to your suggestions, we made several important revisions. Specifically, we clarified and expanded the Materials and Methods section, incorporated additional discussion points on urban greening, and updated the Supporting Information files to improve transparency and reproducibility. Figures 1, 2, 3, and 6 were revised in line with PLOS ONE guidelines and copyright policies, with updated legends providing more comprehensive detail.

During a review of spatial residual autocorrelations, we also identified an error in the multivariate abundance models. This issue has now been corrected in the Results and Discussion sections. Although the correction modified the ranking in the variable importance plots, it did not alter the overall interpretation or conclusions of the study.

In compliance with the editorial requirements, we also made the following clarifications:

- **Financial disclosure**: The funders had no role in study design, data collection and analysis, decision to publish, or preparation of the manuscript.
- **GBIF dataset**: The dataset shared on GBIF (<https://www.gbif.org/dataset/8e52f35a-2522-4865-8361-5c249310a7cf> or <https://doi.org/10.15468/4qafbu>) consists solely of raw, open-access occurrence records of *Aedes albopictus* collected during our field surveys. These data were not peer-reviewed and have not been formally published in any journal or conference proceedings. The GBIF submission serves only as a public repository to support open data practices, and does not constitute prior publication of the analyses, interpretations, or conclusions presented in this manuscript.
- **Data and code availability**: All data are available through the above GBIF repository (<https://www.gbif.org/dataset/8e52f35a-2522-4865-8361-5c249310a7cf> or <https://doi.org/10.15468/4qafbu>) , and the statistical analysis code is provided at ([https://archive.softwareheritage.org/swh:1:dir:a1c83c4be076b5645d1834265219d4f2e6e5b544;origin=https://github.com/ptaconet/modeling_vector_mtp;visit=swh:1:snp:741ed0f1af295a2a2ef477b310cda3f0faa3f1a0;anchor=swh:1:rev:11de6e23243904d7fa7a21d9d6ade29486d06349](https://archive.softwareheritage.org/swh:1:dir:a1c83c4be076b5645d1834265219d4f2e6e5b544;origin=https:/github.com/ptaconet/modeling_vector_mtp;visit=swh:1:snp:741ed0f1af295a2a2ef477b310cda3f0faa3f1a0;anchor=swh:1:rev:11de6e23243904d7fa7a21d9d6ade29486d06349)).
- **Figure 1**: This figure has been replaced with an original image created by the authors, fully compliant with the CC BY 4.0 license.

This response package is organized as follows: (1) a summary of additional corrections made to the manuscript, (2) clarifications in response to journal-specific requirements, and (3) point-by-point replies to the comments from Reviewer 1 and Reviewer 2.

We hope that these revisions satisfactorily address all concerns and further improve the manuscript. We remain grateful for your careful evaluation and constructive feedback.

**Sincerely,**
Colombine Bartholomée
on behalf of all co-authors

Table of Contents

[Additional changes	2](#_Toc209792104)

[Journal Requirements	4](#_Toc209792105)

[Reviewer #1:	7](#_Toc209792106)

[Reviewer #2:	7](#_Toc209792107)

# Additional changes

When assessing spatial residual autocorrelation in the Random Forest abundance models (Reviewer 2, Comments 4 & 18), we discovered that, during recursive feature elimination, some variables essential for accounting for autocorrelation had been excluded. We therefore reintroduced these variables into the final model (namely, cumulative rainfall during the six weeks preceding sampling and maximum hourly relative humidity within the 24 hours prior to sampling). This adjustment altered the ranking in the variable importance plot for abundance but did not change the overall interpretation of the results. Accordingly, we revised:

- the Abstract:
  - Lines 40-42: “*While urban vegetation had a limited effect on Ae. albopictus presence, the average patch size, and the percentage of area covered by low vegetation were among the most important predictors of abundance.”*
  - Lines 44-46: *“The most important predictors of abundance were the average patch size of low vegetation, the maximum hourly temperatures during sampling, and the length of roads.”*
- the *Results* section, specifically the “Abundance Model” paragraph within “Multivariate Analysis”
  - Lines 560-580:”*The most contributing variables to* Ae. albopictus *abundance comprised a combination of microclimatic, weeks-lagged meteorological, vegetation and other land cover variables. The first contributing variable was the average size of low-vegetation patches within a 50 m radius buffer around traps (Fig 6A). Abundance increased markedly for areas between 0 and 120 m², plateaued up to 237 m², and subsequently decreased slightly (Fig 6B). The second most important variable was the local maximum hourly temperature during sampling. A strong positive correlation with abundance was observed between 24°C and 38.7°C, followed by a negative correlation beyond 40.6°C. The third key variable was the total length of road edges within a 250 m buffer around traps, with a strong negative correlation between 8230 m and 11 000 m. The fourth contributor was the percentage of low vegetation within a 250 m buffer, which showed a positive effect between 10% and 23%, beyond which it had no observable effect. The fifth and sixth contributors were the cumulated rainfall in the sixth week before sampling and the weekly GDD between the sampling day and two weeks prior, both of which were positively associated with mosquito abundance. The average wind speed between the day of sampling and five weeks before had no effect on abundance between 3.7 m/s and 4.3 m/s, but showed a negative correlation beyond this range. Finally, the last two contributors were the average patch size of roads within a 250 m buffer and the maximum hourly relative humidity during the 24 hours preceding sampling, both of which were slightly negatively correlated with mosquito abundance. The abundance model demonstrated good overall accuracy, except for high abundance cases (>11 mosquitoes, Mean Absolute Error (MAE)=8.00, S8A Fig).*’
  - Figure 6 has been updated to reflect the revised results and has been uploaded as part of the revised submission.
- the *Discussion* section
  - Lines 587 and 589: “*This study examined, for the first time in France, the impact of urban vegetation on the presence and abundance of Ae. albopictus, showing a minimal influence on the presence and a positive association with the abundance*”
  - Lines “597-598”: “*Regarding the abundance, urban vegetation demonstrated a positive effect and emerged as one of the four most influential predictors*”
  - Lines 600-601: “*In our study, the first vegetation metric influencing abundance was the average size of low-vegetation patches within a 50 m buffer radius.* “
  - Lines 623-624: “*The other two land cover variables, the third and eighth contributors to abundance, are road metrics within a 250 m radius buffer around traps*. “
  - Lines 648 – 649: “*Local hourly temperatures emerged as the primary determinant of Ae. albopictus presence and second most influential predictor of its abundance*.”
  - Lines 655-657; “*The third variable influencing the presence of Ae. albopictus was the weekly cumulated rainfall recorded six weeks before sampling, which also ranked as the fifth most important predictor of abundance*.”
  - Lines 667-668: “*The sixth most influential variable of abundance was the weekly GDD during the two weeks preceding sampling.”*
  - Lines 671 and 674: [50]. “*This baseline corresponds to the average annual temperature required for the spread of Ae. albopictus [92]. One of the last variables influencing abundance is the mean daily wind speed during the five weeks preceding sampling*.”
- Supplementary Figure S8 has been updated to reflect the revised results and has been uploaded as part of the revised submission.
- The files 6 and 7 related to the code for the multivariate model have been updated, as reflected in the publicly accessible code repository ([https://archive.softwareheritage.org/swh:1:dir:a1c83c4be076b5645d1834265219d4f2e6e5b544;origin=https://github.com/ptaconet/modeling_vector_mtp;visit=swh:1:snp:741ed0f1af295a2a2ef477b310cda3f0faa3f1a0;anchor=swh:1:rev:11de6e23243904d7fa7a21d9d6ade29486d06349](https://archive.softwareheritage.org/swh:1:dir:a1c83c4be076b5645d1834265219d4f2e6e5b544;origin=https:/github.com/ptaconet/modeling_vector_mtp;visit=swh:1:snp:741ed0f1af295a2a2ef477b310cda3f0faa3f1a0;anchor=swh:1:rev:11de6e23243904d7fa7a21d9d6ade29486d06349)).

# Journal Requirements

When submitting your revision, we need you to address these additional requirements.

1. Please ensure that your manuscript meets PLOS ONE's style requirements, including those for file naming. The PLOS ONE style templates can be found at

https://journals.plos.org/plosone/s/file?id=wjVg/PLOSOne_formatting_sample_main_body.pdf and

<https://journals.plos.org/plosone/s/file?id=ba62/PLOSOne_formatting_sample_title_authors_affiliations.pdf>

The manuscript has been carefully reviewed to ensure full compliance with all PLOS ONE style requirements, including file naming conventions, and any necessary adjustments have been made.

2. Please note that PLOS ONE has specific guidelines on code sharing for submissions in which author-generated code underpins the findings in the manuscript. In these cases, we expect all author-generated code to be made available without restrictions upon publication of the work.

Please review our guidelines at https://journals.plos.org/plosone/s/materials-and-software-sharing#loc-sharing-code and ensure that your code is shared in a way that follows best practice and facilitates reproducibility and reuse.

The code has been made available as indicated in the Methods section (last sentence: “*All scripts are available on Software Heritage [87]*.”). Our code is shared in accordance with best practice, facilitating reproducibility and reuse.

3. Thank you for stating the following financial disclosure:

“This work is part of the V2MOC project, led by FF and funded by the Occitanie Region as part of the Défi Clé RIVOC (https://www.umontpellier.fr/universite/projets-emblematiques/defis-cles-de-la-region-occitanie). CB received a doctoral scholarship from the Défi Clé RIVOC of the Occitanie Region and the University of Montpellier.”

Please state what role the funders took in the study. If the funders had no role, please state: "The funders had no role in study design, data collection and analysis, decision to publish, or preparation of the manuscript." If this statement is not correct you must amend it as needed.

Please include this amended Role of Funder statement in your cover letter; we will change the online submission form on your behalf.

This statement regarding the role of the funders has been added to the cover letter: *'The funders had no role in study design, data collection and analysis, decision to publish, or preparation of the manuscript*.'

4. We noted in your submission details that a portion of your manuscript may have been presented or published elsewhere:

“Field data have been published on GBIF: https://www.gbif.org/dataset/8e52f35a-2522-4865-8361-5c249310a7cf”

Please clarify whether this [conference proceeding or publication] was peer-reviewed and formally published. If this work was previously peer-reviewed and published, in the cover letter please provide the reason that this work does not constitute dual publication and should be included in the current manuscript.

We thank the editor for this request for clarification. The dataset we shared on GBIF represents raw, open-access occurrence records of *Aedes albopictus* collected during our field surveys. These records were not peer-reviewed and have not been formally published in any journal or conference proceedings. The GBIF submission serves solely as a public data repository to comply with open data practices and does not constitute prior publication of the analyses, interpretations, or conclusions presented in the current manuscript. Therefore, the content of the present manuscript, including statistical analyses, integration with environmental and landscape variables, and ecological interpretation, is entirely original and has not been published elsewhere.

This statement has been added to the the reponse letter.

5. Please note that your Data Availability Statement is currently missing the repository name and direct link to access each database. If your manuscript is accepted for publication, you will be asked to provide these details on a very short timeline. We therefore suggest that you provide this information now, though we will not hold up the peer review process if you are unable.

We apologize for this oversight. As noted in the manuscript (lines 179, ref. 46), both the entomological data and the primary environmental and climatic datasets are publicly available via GBIF and can be accessed directly at the following link: <https://doi.org/10.15468/4qafbu>.

6. We note that Figure 1 in your submission contain map/satellite images which may be copyrighted. All PLOS content is published under the Creative Commons Attribution License (CC BY 4.0), which means that the manuscript, images, and Supporting Information files will be freely available online, and any third party is permitted to access, download, copy, distribute, and use these materials in any way, even commercially, with proper attribution. For these reasons, we cannot publish previously copyrighted maps or satellite images created using proprietary data, such as Google software (Google Maps, Street View, and Earth). For more information, see our copyright guidelines: http://journals.plos.org/plosone/s/licenses-and-copyright.

We require you to either (1) present written permission from the copyright holder to publish these figures specifically under the CC BY 4.0 license, or (2) remove the figures from your submission:

1) You may seek permission from the original copyright holder of Figure 1 to publish the content specifically under the CC BY 4.0 license.

We recommend that you contact the original copyright holder with the Content Permission Form (http://journals.plos.org/plosone/s/file?id=7c09/content-permission-form.pdf) and the following text:

“I request permission for the open-access journal PLOS ONE to publish XXX under the Creative Commons Attribution License (CCAL) CC BY 4.0 (http://creativecommons.org/licenses/by/4.0/). Please be aware that this license allows unrestricted use and distribution, even commercially, by third parties. Please reply and provide explicit written permission to publish XXX under a CC BY license and complete the attached form.”

Please upload the completed Content Permission Form or other proof of granted permissions as an ""Other"" file with your submission.

In the figure caption of the copyrighted figure, please include the following text: “Reprinted from [ref] under a CC BY license, with permission from [name of publisher], original copyright [original copyright year].”

2) If you are unable to obtain permission from the original copyright holder to publish these figures under the CC BY 4.0 license or if the copyright holder’s requirements are incompatible with the CC BY 4.0 license, please either i) remove the figure or ii) supply a replacement figure that complies with the CC BY 4.0 license. Please check copyright information on all replacement figures and update the figure caption with source information. If applicable, please specify in the figure caption text when a figure is similar but not identical to the original image and is therefore for illustrative purposes only.

The following resources for replacing copyrighted map figures may be helpful:

USGS National Map Viewer (public domain): http://viewer.nationalmap.gov/viewer/

The Gateway to Astronaut Photography of Earth (public domain): http://eol.jsc.nasa.gov/sseop/clickmap/

Maps at the CIA (public domain): https://www.cia.gov/library/publications/the-world-factbook/index.html and https://www.cia.gov/library/publications/cia-maps-publications/index.html

NASA Earth Observatory (public domain): http://earthobservatory.nasa.gov/

Landsat: http://landsat.visibleearth.nasa.gov/

USGS EROS (Earth Resources Observatory and Science (EROS) Center) (public domain): http://eros.usgs.gov/#

Natural Earth (public domain): http://www.naturalearthdata.com/

Thank you for your helpful suggestion**.** In response, we have removed the Google Maps image and replaced it with our own photographs of the study areas. To ensure proper attribution, we have also included a reference to OpenStreetMap in the figure caption: *“Map data © OpenStreetMap contributors, licensed under ODbL.”* (Lines 151-152).

7. If the reviewer comments include a recommendation to cite specific previously published works, please review and evaluate these publications to determine whether they are relevant and should be cited. There is no requirement to cite these works unless the editor has indicated otherwise.

# Reviewer #1:

Dear Authors, this work needs to be a great efforts during the data collection and evaluation of the collected data. manuscript techically sound but not easy to follow during reading. therefore need to be shortened some parts for easy to follow.

We thank Reviewer 2 for the comment regarding manuscript length. We have condensed the text where possible, but certain sections—particularly on the creation of the land cover map and the extraction of socio-economic data (Comment 2)—needed to be expanded to ensure reproducibility. These additions provide necessary detail for transparency and clarity, while the rest of the manuscript remains concise.

Maybe PCA is another option for evaluating the parameters contribution presence and abundance.

We thank the reviewer for this interesting suggestion. Principal Component Analysis (PCA) is indeed a valuable method for dimensionality reduction and for summarizing correlated predictors. However, in our study, the aim was not to reduce predictors into composite axes but rather to evaluate the direct contribution of specific environmental and landscape variables (e.g., vegetation cover, building density, meteorological conditions) to the presence and abundance of *Ae. albopictus*. Using PCA would have made the interpretation of individual variable effects more difficult, since biological meaning is less straightforward once predictors are combined into principal components.

To address multicollinearity among predictors, we instead applied variance inflation factor (VIF). This allowed us to retain explanatory variables while ensuring model robustness, and at the same time to quantify the effect sizes of specific predictors that are directly relevant to mosquito ecology and urban planning.

# Reviewer #2:

Overall, this is an interesting and timely study examining how urban vegetation affects the presence and abundance of *Aedes albopictus* in Montpellier, France. The topic is important, bridging urban planning, vector ecology, and public health. The research objectives are clearly stated, and the study provides useful insights that could inform future management of urban green spaces and vector control strategies.

Technical Soundness and Data Support

The study is generally sound, and the data collected are extensive. The methodology is mostly robust, but some clarifications would help fully support the conclusions:

Comment 1: The exact temporal lags used for meteorological and environmental variables should be specified more clearly.

Please see our answer to comments 23-24 & 26.

Comment 2: It is unclear whether datasets with different spatial resolutions (e.g., land cover, population, infrastructure) were harmonised or simply intersected with trap locations and buffers.

We thank the reviewer for this important comment. We confirm that datasets with different spatial resolutions were harmonised prior to analysis. Specifically, we combined three datasets to produce a consistent land-cover map (0.5 m resolution) used in subsequent analyses (see Supplementary Table 2):

- Vegetation dataset: fine-scale vegetation map of Montpellier (Montpellier Méditerranée Métropole), generated using a hybrid approach combining deep learning image processing and photo-interpretation of Pléiades satellite imagery with a LiDAR-derived elevation model. This vector dataset included polygons > 5 m².
- Building dataset: French national building database (Base de données nationale des bâtiments, BDNB), created by cross-referencing ~20 public datasets. This vector dataset has < 1 m precision.
- LULC dataset: Copernicus Urban Atlas (European Union), vector dataset with polygons > 2,500 m².

Land-cover harmonisation workflow:

- All three datasets were rasterised at 0.5 m spatial resolution.
- Empty cells (NA values) in the vegetation raster were filled using values from the building dataset, resulting in a “vegetation + building” layer.
- Remaining NA cells were filled with values from the LULC dataset.
- Land-cover classes were merged into five categories:
  - low vegetation (< 3 m height), high vegetation (> 3 m height), from the vegetation dataset,
  - buildings, from the buiding dataset,
  - roads, and others, from the LULC dataset.

The R code used for this process is openly available (file 1_prepare_data.R in reference 87 of the manuscript).

Spatial analyses were then performed to extract, for each buffer around traps (20–250 m radii), the percentage of area, total edge length, number of patches of each land-cover class, and the Shannon index of landscape diversity.

The manuscript (*Methods* section, § *Fine-scale vegetation and land cover data*) was modified to make it clearer:

*“A harmonised land-cover map of Montpellier was generated at 0.5 m resolution by combining three complementary datasets (see Supplementary Table S2): (i) the fine-scale vegetation dataset of Montpellier (Montpellier Méditerranée Métropole, 2019), which distinguishes vegetation patches using deep learning and photo-interpretation of Pléiades satellite imagery and a LiDAR-derived elevation model; (ii) the French national building database (Base de données nationale des bâtiments, BDNB); and (iii) the Copernicus Urban Atlas. Following rasterisation and integration of these sources, five land-cover categories were retained (Fig 1B): buildings, roads, low vegetation (< 3 m height), high vegetation (> 3 m height), and “others” (including railways, pathways, industrial areas, and sports facilities).[...]”*

Regarding demographic and social data, we used two complementary databases:

- Montpellier fine-scale population dataset: population counts from each sub-municipal IRIS unit (~2,000 inhabitants) were redistributed proportionally to the living area of individual land plots. The resulting dataset provides georeferenced points (X, Y) located at the centre of significant buildings, each associated with a population value (rounded to the nearest whole number).
- Filosofi database (INSEE, 200 m resolution): used to extract household standard of living and household age structure.

For each buffer area, we summed population counts (from the point dataset), number of poor households, and number of buildings of different ages (from the Filosofi grid) across all intersecting spatial units.

The manuscript (*Methods* section, § *Social and demographic data*) was revised as follows:

*“Number of buildings of different ages and number of low-income households (2019) were obtained from the Filosofi database of the French Institut National de la Statistique et des Études Économiques (INSEE), provided as a 200 m resolution grid. For each buffer area, values of intersecting grid cells were summed. Human population data were obtained from the Montpellier fine-scale population dataset (Supplementary Table S2), in which IRIS-level population counts (~2,000 inhabitants per unit) were redistributed across land plots in proportion to living area and assigned to the centroid of significant buildings. Population counts were then aggregated within the same four buffer zones (20, 50, 100, and 250 m radii).”*

Comment 3: No information is provided on potential vector control interventions, such as municipal insecticide spraying or larvicide treatments, which could affect mosquito presence and abundance. Clarifying whether these were monitored or considered in the analysis would strengthen the study.

Please see our answer to Comment 17.

Statistical Analysis

The statistical approach appears rigorous and appropriate. The two-stage modelling strategy—using GLMMs for bivariate screening and random forest models for multivariate analysis—is well justified and accounts for potential non-linear relationships and interactions. Including both spatial and temporal cross-validation adds robustness.

Comment 4: However, the supplementary materials could include more details to enhance reproducibility and transparency, such as: Specifications of the GLMM and random forest models (e.g., distributions, link functions, hyperparameters), Cross-validation results and model diagnostics, Residual spatial autocorrelation analyses, Sensitivity analyses regarding buffer sizes and temporal lags.

Please see our answer to Comment 18.

Presentation and Writing

The manuscript is generally clear and written in standard English. The structure is logical, and the main concepts are understandable. Minor improvements could make it even smoother:

Comment 5: Improve transitions in the Introduction between urbanisation and re-greening.

Please see our answer to comment 14.

Comment 6: Clarify certain phrases in Materials & Methods (e.g., “were vegetated” → “were vegetated areas”; precise description of 24- vs 48-hour temporal lags; details of microclimate and rainfall measurements).

Please see our answers to Comments 20-24.

Comment 7: Ensure consistent definitions for land cover metrics and statistical indices.

Please see our answers to Comment 21-22.

Additional Comments and Recommendations

Comment 8: The Discussion is strong and compares findings with previous studies well, but it could more explicitly inform practical urban planning, such as optimal green area dimensions, vegetation management strategies, and balancing ecological benefits with vector-related risks.

Please see our answer to Comment 34.

Comment 9: Recording vegetation type (plant species) in future studies could help identify which species influence mosquito presence and abundance, providing actionable guidance for urban planners.

We thank the reviewer for this insightful suggestion. We agree (as discussed in § *Limitations, perspectives and conclusion, l.717-724*) that identifying vegetation at the species level could provide valuable information on which plant taxa influence mosquito presence and abundance, and thereby offering practical guidance for urban greening strategies. While the present study focused on structural vegetation metrics (e.g., cover, patch size, and diversity), we are addressing the question of plant species use by *Ae. albopictus* within the same research project by analyzing plant DNA in mosquito sugar meals. These complementary data will help to link vegetation composition with mosquito feeding behaviour and abundance, and we plan to integrate these findings in future work.

Comment 10: It would be valuable in future studies to examine whether arbovirus cases occurred in areas corresponding to environmental variables positively or negatively associated with mosquito abundance to reinforce the public health relevance.

We appreciate this constructive suggestion. We agree that linking mosquito abundance with arbovirus case occurrence would further strengthen the public health relevance of our findings. While the present study focuses on environmental drivers of *Ae. albopictus* presence and abundance, within the same research project we have developed transmission models for dengue, chikungunya, and Zika viruses. These models integrate mosquito abundance and age structure to estimate the basic reproduction number (R₀). A manuscript reporting these results, which form part of the first author’s PhD thesis, is currently in preparation and will soon be submitted for publication. Together, these complementary studies will provide both ecological and epidemiological insights into the role of urban environments in shaping arbovirus transmission risk.

Please note that we discussed in the manuscript that presence and/or abundance of mosquitoes are necessary but not sufficient data to address risk transmission (§ *Limitations, perspectives and conclusion, l.714-717*).

Comment 11: Since the study is based on a single year of sampling, additional years of data would allow more robust conclusions.

We thank the reviewer for this important comment. We acknowledge that our adult mosquito dataset covers a single sampling season, which may limit the generalisability of inter-annual patterns (as discussed in § *Limitations, perspectives and conclusion, l.693-695, l. 713-714, l. 735-738*). Extending adult collections over multiple years was unfortunately not feasible due to financial and logistical constraints. However, within the same project we conducted weekly egg collections with ovitraps over multiple consecutive years. A similar analysis, with similar results to those presented here, has been performed using these long-term oviposition data, and a manuscript is currently in preparation. We believe that, together, these complementary datasets (multi-year eggs and single-season adult collections) will provide a more comprehensive understanding of *Ae. albopictus* population dynamics in urban environments.

Conclusion

This study makes a valuable contribution to understanding urban vector ecology and the role of vegetation in shaping mosquito populations. With clarifications and additional details on statistical methods and environmental factors, the manuscript could provide strong, actionable insights for urban planning and vector management.

**ABSTRACT**

Comment 12: **Line 31**: I was wondering why the term *“impervious”* is used in the context of urban environments. Is it related to the *imperviousness* variable, in the sense that a more impervious environment indicates a higher degree of urbanisation?

We thank the reviewer for pointing out this potential ambiguity. Indeed, we used the term “impervious” in reference to the imperviousness of land cover, which is a widely applied proxy for the intensity of urbanization. In our study, the “impervious city center” refers to the most urbanized part of the city, characterized by a high proportion of sealed surfaces (e.g., asphalt, concrete, buildings) and very limited vegetation. To avoid confusion, we will clarify this wording in the Abstract and Methods.

Revised text in Abstract:

“*From May to October 2023, adult mosquitoes were collected monthly in urban parks, residential areas, and the highly impervious city center using BG-Pro traps with odor and CO₂ attractants.”*

Revised text in Methods (l.138-140):

*“[...] and four highly impervious (i.e., areas of the city center dominated by sealed surfaces such as concrete and asphalt) area (IMP)”*

Moreover, the expressions “densely built impervious areas”, “impervious built-up areas”, and “impervious built areas” were changed to “*highly impervious areas*” throughout the manuscript:

Comment 13: **Line 32**: It may be helpful to specify more clearly how on-site microclimate was measured.

We thank the reviewer for this suggestion. In the original version, our description of the microclimate measurements was too general. Microclimate data were recorded on-site using Hygrobutton data-loggers, which continuously monitored air temperature and relative humidity at each trapping location. We will revise the text accordingly for greater clarity.

Revised text in Methods (lines 32):

“Microclimate data (air temperature and relative humidity) were recorded on-site at each trap location using Hygrobutton data-loggers”

**INTRODUCTION**

Comment 14: The transition between urbanisation and its description, and the subsequent discussion on re-greening, is not entirely smooth.

Overall, the introduction conveys the main concepts effectively, and the study objective is clear.

We thank the reviewer for this comment. We agree that the transition between the description of urbanization impacts and the introduction of Nature-Based Solutions (NBS) could be smoother. To address this, we have revised the paragraph to explicitly connect the challenges of urbanization with the role of NBS as a mitigation strategy.

Revised text in Introduction:

*“[…] worsening its impact on human health [9].*

*Given these multiple pressures, strategies that simultaneously address environmental and social challenges are increasingly needed. Nature-Based Solutions (NBS), such as urban greening, are promoted to […]”*

Comment 15: **Line 75**: I do not fully understand how *social disparities* are linked to urbanisation. Does urbanisation increase these disparities, which in turn exacerbate the risk of mental health issues? This connection could be explained more explicitly.

We thank the reviewer for this comment. Our intention was to highlight that rapid urbanisation can exacerbate social disparities, for instance through unequal access to green spaces, healthcare, and healthy living conditions. These disparities, in turn, increase vulnerability to stress and mental health disorders. To make this connection clearer, we have revised the text accordingly.

Revised text in Introduction (lines 75-78):

*“Moreover, rapid urbanisation often accentuates social disparities, as access to green spaces, healthcare, and healthy living conditions is not equally distributed across urban populations. These inequalities, together with reduced opportunities for contact with nature, increase vulnerability to stress and mental health disorders [7].”*

**MATERIALS & METHODS**

Comment 16: Overall, the section requires revision to enhance clarity for the reader. It is not entirely clear which temporal lags were used, nor whether there is spatial aggregation of the data, and at what resolution (e.g. land cover). Spatial resolution is reported only for infrastructure age and low-income household data. Was this resolution (200 m grid) maintained, or were datasets at different resolutions (to be specified) intersected with trap locations and buffers, with values then assigned accordingly? This should be clarified.

Please refer to our responses to Comments 23-24 & 26 regarding temporal lags and Comment 2 regarding the spatial resolution of the data.

Comment 17: Also, It is not clear whether potential vector control interventions (e.g. insecticide spraying or larvicide treatments conducted by the municipality in urban green areas) were monitored or considered during the study period. Such interventions could strongly influence mosquito abundance and presence, and therefore it would be important to clarify whether information on disinfestation activities was collected and how this factor was handled in the analysis.

**We thank the reviewer for this valuable and insightful comment.** In France, adulticide treatments are applied only in response to **confirmed cases of dengue, chikungunya, or Zika virus**, within a 150m radius from the case location, at night, using pyrethroids at very low concentrations (0.5 g/ha), and only outdoors. During 2023, two indigenous dengue cases were reported in Montpellier (between September and October) in the Estanove neighborhood, approximately 700 m from the nearest sampling site. Given the limited treatment radius, these interventions would not have affected our collections. No adulticide treatments were conducted within our study areas.

We also contacted the organizations responsible for larvicide and adulticide applications, and **no interventions were reported in the sampling areas.** Weekly monitoring conducted in parallel revealed no sudden decreases in egg numbers, further supporting the absence of treatment effects. In residential areas**, no individual actions by residents** (e.g., mechanical removal of larval habitats) were observed during our surveys. However, we acknowledge that such resident-driven interventions **may have occurred in near some sampling sites and could not be systematically assessed**, representing a limitation of the study.

Accordingly, we have added a the following description in the Materials & Methods section, § “Environmental data collection and preparation”, (*lines 265-269)*: “*No municipal adulticide or larvicide treatments were implemented in the study areas during the sampling period. No individual larval habitat removal by residents was observed. However, potential actions by residents near some sampling sites could not be systematically assessed, and their effects on mosquito abundance cannot be excluded*.”

Additionally, we have included a sentence in the **Discussion** section (*§****Limitations, perspectives, and conclusions,*** *lines 706-708*) to address this point: *“Finally, as described in the Materials and Methods, no municipal vector control interventions were observed in the sampling areas. Nevertheless, potential actions by residents in houses close to the sampled sites could not be assessed.”*

Comment 18: Additionally, while the general modeling workflow is described, the supplementary materials could be expanded to include more technical details on the statistical analyses. This could include a detailed diagram of the variable selection and modeling workflow, specifications of the GLMM and random forest models (e.g., distributions, link functions, hyperparameters), cross-validation results (spatial and temporal), diagnostics of model performance and residual spatial autocorrelation, and sensitivity analyses regarding buffer sizes and temporal lags. Providing these details would improve transparency, reproducibility, and the reader’s ability to assess the robustness of the results.

We thank the reviewer for this insightful comment. The general modelling workflow diagram (S2 Fig) has been updated to include additional details on the GLMM (distributions, link function, and random effects), Random Forests (recursive feature elimination, hyperparameters such as the number of trees and splitting function), cross-validation results (performance metrics), and residual spatial autocorrelation. Additional information is provided in file_4_Building_Model and files 6_7 on Multivariate_Modeling (reference 87 of the manuscript). Diagnostics for the presence and abundance models are available in S7 and S8 Figs, respectively. Moreover, we have prepared a new S1 Text containing a detailed description of the methods.

No sensitivity analyses were conducted regarding buffer sizes, as the relationship with mosquito presence or abundance sometimes varied depending on the radius, while in other cases, no clear effect was observed. This likely reflects the heterogeneous nature of the urban environment, where the scale of influence of landscape features can differ locally. For temporal lags, we focused those with higher marginal R², which are also consistent with biologically relevant lags identified in previous studies. References to S1 Text have been added in lines 292 and 312.

Comment 19: **Line 137**: Replace “were vegetated” with “were vegetated areas” or “were covered with vegetation”.

Done.

Comment 20: **Lines 168–172**: It is not clear whether one 48-hour trapping session per month was conducted, or whether multiple 48-hour sessions took place within each month. From the results it appears there was one per month per site, which should equal 7 × 15 collections. It is also unclear whether mosquitoes were collected each morning during the 48 hours (i.e. twice per sampling event) or only once after the full 48 hours.

We thank the reviewer for this request for clarification. One 48-hour trapping session was conducted per site per month between May and October 2023. During each 48-hour session, mosquitoes were retrieved every morning, resulting in two collections per session. In total, this corresponds to 7 sites × 6 months × 2 collections = 84 collections. We have revised the Methods text accordingly.

Revised text in Methods (lines 174 – 176):

*“One 48-hour trapping session was conducted per site per month from May to October 2023. During each session, mosquitoes were retrieved each morning, resulting in two collections per session. Mosquitoes were transported at 4°C to the laboratory […]”*

Comment 21: **Lines 192–194**: A description of land cover classes, either in the draft or in the Supplementary Materials, would be useful. The definitions of *percentage of cover*, *total edge length*, and *average patch size* should also be provided, along with details on how they were calculated.

The definitions of land cover classes and landscape metrics are already provided in Supplementary Table 2. To improve clarity, we have now explicitly described these classes and provided definitions of the metrics (percentage of cover, total edge length, and average patch size) in the Methods, while maintaining the detailed listing in the Supplementary Materials.

Revised text in Methods (lines 200 – 205):

*“For each trap location, spatial metrics were computed within circular buffer zones of 20 m, 50 m, 100 m, and 250 m radii. For each land cover category, we calculated the percentage cover (proportion of buffer area occupied by the category), total edge length (sum of the perimeters of all patches of the category), and average patch size (mean area of individual patches). For vegetation categories (low vegetation < 3 m height, high vegetation > 3 m height), we also extracted the number of patches.”*

Comment 22: **Line 195**: Provide a definition of the Shannon index.

To improve clarity, we now provide a concise definition of the Shannon index in the Methods (liens 206-208) and refer to Supplementary Table 2 where we now indicted the formula.

Revised text in Methods:

*“The landscape diversity (Shannon index) was calculated for all buffer zones. This index quantifies diversity by considering both the number of land cover categories and their relative proportions, with higher values indicating more heterogeneous landscapes (see Table S2 for the formula).”*

Comment 23: **Line 204**: The shift from 24 h to 48 h lags is unclear. Perhaps it would be clearer to write: *“for 24-hour periods starting from 48 hours prior to the beginning of each sampling session until its end”*.

Agree. The text was revised accordingly.

Comment 24: **Line 208**: Similarly, I would suggest: *“24-hour periods prior to and during the sampling sessions …”*. Does “as for datalogger data” mean that microclimate was measured with the datalogger and values were taken exactly as recorded?

We thank the reviewer for pointing this out. To clarify, precipitation was not measured with the data loggers but obtained from the nearby weather station. Precipitation data were analysed using the same weekly and multi-week summary approach as other meteorological variables, as well as using 24-hour summaries like the microclimate data, because precipitation may influence mosquito activity and therefore sampling. We have revised and moved the sentence to indicate that precipitation was also aggregated over 24-hour periods in the same temporal structure as the datalogger variables, while justifying the approach and specifying the source. Additionally, we have now clearly separated microclimate data (collected using data loggers) and meteorological data (obtained from weather stations) into distinct sections.

Revised text in Methods (moved at the end of the §, lines 233 to 236):

*“Precipitation data from the Château d'Ô station were also aggregated over 24-hour periods prior to and during each sampling session, following the same temporal aggregation approach used for microclimate data from the dataloggers because precipitation may influence mosquito activity and therefore sampling.”*

Comment 25: **Lines 206–209**: Which rainfall dataset was used—the hourly data from the Observatoire Départemental de l’Eau et de l’Environnement (ODEE) station, or from the datalogger?

Precipitation data were obtained exclusively from the nearest Observatoire Départemental de l’Eau et de l’Environnement (ODEE) weather station, not from the dataloggers. We have clarified this in the Methods (please see our answer to previous comment).

Comment 26: **Line 215**: *“All weather data were aggregated for the sampling week and each of the six weeks before sampling”*. Does this mean the temporal resolution is weekly, or were different lags created (e.g. weekly mean temperature, two-week mean, daily, two-day, etc.)? Please clarify how the temporal aggregation was performed for the models.

To clarify, weather data were summarized at weekly and multiple-week resolutions. For each sampling event, we considered not only the sampling week (W0) itself but also all possible lag periods between W–6 and W0, with durations ranging from one to seven consecutive weeks. This approach allowed us to capture both short- and longer-term weather effects in cross-correlation maps. We have revised the text to reflect this procedure more clearly.

Revised text in Methods (lines 228 – 233):

“For each sampling event, weather data were aggregated at a weekly resolution for the sampling week (W0) and for all possible lag periods between W–6 and W0. These lag periods ranged in duration from one to seven consecutive weeks (e.g., W–2 only, W–4 to W–2, W–6 to W0). Rainfall and GDD were summed, while temperature, humidity, and wind speed were averaged over each lagged window.”

In addition, the same clarification was made for air quality data (l.249-251):

“As for weather data, air quality data were aggregated at a weekly resolution for the sampling week (W0) and for all possible lag periods between W–6 and W0, by averaging the daily concentrations of each pollutant over these windows.”

Comment 27: **Lines 218–220**: This reads more like material for the Introduction or Discussion. I would recommend moving the justification for the variable choice after its description and derivation.

In the revised manuscript, we have retained a concise justification for including atmospheric pressure but moved it to precede the variable description, in line with the reviewer’s recommendation. This keeps it brief, avoids sounding like a literature review, and directly links rationale and measurement.

Revised text in Methods (lines 237 – 244):

*“As atmospheric pressure can influence insect flight and activity [51–53], we evaluated its effect on Ae. albopictus. For this purpose, the minimum (lowest recorded value), maximum…”*

Comment 28: **Lines 228–231**: Same comment as for lines 218–220.

Same as above.

Revised text in Methods (lines 245-246):

*“As air pollution can influence insect behaviour and abundance[54-56], we evaluate the effect of air quality data on Ae. Albopictus. For each trap location…”*

Comment 29: **Lines 241–244**: Please specify the years for these data.

Data on infrastructure age and the number of low-income households are from 2019 and were published in 2023. This information has now been indicated in the manuscript (line 256).

Comment 30: **Line 246**: Are the population data from 2016? Are more recent datasets available?

The population data are from 2020. This has been specified in Material & Methods, *§Social and demographic* data, line 260, and in Table S2.

**RESULTS**

In general, the results are presented clearly and logically.

Comment 31: **Line 334 – Table 1**: Should be written *Cs. longiareolata* and *Cs. annulata*.

Agree. Modified accordingly.

Comment 32: **Line 427**: Typo: “associtaion” → “association”.

Corrected.

Comment 33: **Line 476**: Since population density is discussed here, the Materials and Methods should specify how it was calculated.

Done. § *Social and demographic data* in the *Methods* section was rewritten (lines 253 – 263).

**DISCUSSION**

Comment 34: Overall, the Discussion is solid and compares the findings with previous studies. However, there is limited effort to define how the results could inform practical urban planning (e.g. optimal dimensions of green areas, vegetation management in residential zones, trade-offs between ecological benefits and vector-related risks). Strengthening this aspect would considerably enhance the impact of the paper.

We thank the reviewer for this constructive comment. We fully acknowledge the importance of linking our findings to practical urban planning applications. In response, we have expanded the Discussion to include specific recommendations based on both the literature and our results. These include recommendations on the size and spatial arrangement of vegetation patches (e.g., small patches at least 50 m from residential areas, discontinuous corridors, diverse vegetation types) and management practices that may reduce mosquito proliferation (e.g., weekly removal of potential breeding sites, avoidance of naturally larval-hosting plants, maintenance of less dense shrubbery). However, we also note that these suggestions remain preliminary. Additional data collection is needed to validate and refine these recommendations, ensuring robustness and generalizability across different urban contexts. This addition has been included at the end of the revised paragraph in the Discussion (Lines 726-738):

“*Another possible implication for urban planning would be to promote the establishment of small vegetation patches located at least 50 m away from residential areas. These patches could be separated by permeable soil or arranged as discontinuous corridors composed of diverse vegetation types (e.g., grass, scattered trees). Vegetation management and good gardening practices could further limit mosquito proliferation, for instance by emptying potential breeding sites at least once a week, avoiding plants that naturally serve as larval habitats (e.g., bromeliads [104]), and reducing features such as bamboo nodes [29]. Maintaining less dense shrubs could help reduce the number of available resting sites [35]. Such practices would allow urban green spaces to retain their ecological and recreational functions while reducing mosquito abundance. However, these suggestions are currently limited to the local context of Montpellier and would require additional sampling, including in other locations, to ensure more robust and generalizable recommendations*.”

**FIGURES**

Comment 35: **Figure 1**: Panel B lacks a scale bar, and a north arrow would be useful. In addition, the outline colours of sampling environments are difficult to distinguish from the land cover map, which uses similar colours.

**Thank you very much for these helpful comments.** We have improved the visibility of the sampling environments by adjusting the outline colors and thickening the lines (see the updated figure below). Additionally, we have made the scale bar more prominent and added a north arrow to clearly indicate orientation.

Comment 36: **Figure 6**: The legend does not indicate the meaning of the asterisk.

There are no asterisks in Figure 6. However, asterisks in Figures 2 and 3 now have their meaning clearly indicated in the respective figure legends.

**FUTURE PROSPECTS AND GENERAL THOUGHTS**

This is a very interesting study, particularly in the framework of Agenda 2030 and in reconsidering urban land use. In the context of rising temperatures and increasing urbanisation, the study may pave the way for targeted investigations aimed at integrating documented scientific evidence into urban planning. At present, such planning is often undertaken without considering the loss of ecosystem services or the implications of invasive species that may thrive in anthropogenic environments, particularly those of public health relevance.

Comment 37: However, there are issues concerning the Materials and Methods section, and, as also noted in the text, the draft relies on a single year of sampling. I encourage the authors to consider publication only after collecting at least one additional year of data, which would strengthen the analyses, allow more robust conclusions, and provide a solid foundation for concrete contributions to urban green planning.

We thank the reviewer for this thoughtful comment. We fully agree that multi-year datasets provide stronger foundations for ecological inference, particularly for informing urban planning. Unfortunately, extending adult sampling over additional years was not possible within the scope of this project due to resource constraints. To mitigate this limitation, we performed intensive monthly adult collections over six months, complemented by fine-scale environmental and landscape data, which allowed us to capture robust associations between urban characteristics and Ae. albopictus abundance. In parallel, we have conducted weekly ovitrap monitoring over multiple consecutive years, and a complementary manuscript based on these data is currently in preparation. We therefore believe that, despite the single-year adult dataset, the present study makes a timely and relevant contribution by demonstrating how structural features of the urban environment shape mosquito populations.

Comment 38: I also recommend consulting the following preprint, which addresses a very similar and directly relevant topic and may provide valuable insights for this study and future work:
*Mercat M, Bartholomée C, Fournet F, Alcover Amengual M, Bourquia M, Bouhsira E, Cornel A, Fernandez Cassi X, Fontenille D, Ibáñez-Justicia A, Marti R, Moiroux N, Niang EH, Smith W, Spitzen J, Visser TM, Koenraadt CJM, Simard F. Green cities and the risk for vector-borne disease transmission for humans and animals: a scoping review**. bioRxiv 2025.04.01.646559; doi:* [*https://doi.org/10.1101/2025.04.01.646559*](https://doi.org/10.1101/2025.04.01.646559?utm_source=chatgpt.com)

We thank the reviewer for pointing out this recent preprint, which indeed addresses themes highly relevant to our study. We are among the co-authors of this work and have already cited it in both the Introduction and Discussion sections of the present manuscript (ref 24), as it provides important context on the links between urban greening and vector-borne disease risk. We fully agree that this scoping review complements our findings and will help guide future research directions in this field. We have also updated the reference to include the DOI.

Comment 39: It would also have been valuable to record not only microclimate but also vegetation type at each sampling site (i.e. plant species present). In future studies, it could be investigated which plant species have the greatest influence on mosquito presence and abundance, as suggested by previous work (e.g. Samson et al. 2014). Such knowledge could provide urban planners and green-space managers with practical guidance on plant species selection to reduce mosquito populations.

Please see our answer to Comment 9.

Comment 40: Finally, in future studies, it would be interesting to evaluate whether arbovirus cases occurred in areas characterised by variables positively associated with mosquito abundance and presence identified in this study, or conversely in areas with features negatively associated with these outcomes.

Please see our answer to Comment 10.
